# CRISPR-mediated gene editing to rescue haploinsufficient obesity syndrome

**DOI:** 10.1007/s13238-019-0635-y

**Published:** 2019-05-23

**Authors:** Zhifeng Wang, Liu Yang, Shen Qu, Chao Zhang

**Affiliations:** 1 Translational Medical Center for Stem Cell Therapy and Institute for Regenerative Medicine, Shanghai East Hospital, Shanghai Key Laboratory of Signaling and Disease Research, School of Life Sciences and Technology, Tongji University, Shanghai, 200092 China; 2 Research & Development Department, Sinoneural Cell and Gene Engineering Holdings Co., Ltd., Shanghai, China; 3 Department of Endocrinology and Metabolism, National Metabolic Management Center, Shanghai Tenth People’s Hospital, School of Medicine, Tongji University, Shanghai, 200092 China

CRISPR and adeno-associated virus are becoming powerful tools to remedy genetic disorders in somatic cells of adulthood. A recent study published in *Science* (Matharu et al., 2019) safely targeted the non-coding genomic region of *Sim1* and *MC4R* with rAAV packed with CRISPRa, and successfully rescued the obesity syndrome caused by haploinsufficiency in a murine model, which shed light on their potential therapeutic applications in the future (Matharu et al., 2019).

CRISPR-Cas9, first described as an adaptive immune system in bacteria, has now been developed as RNA-guided endonucleases for genome editing. As the fourth approach after meganucleases, Zinc-finger nucleases (ZFN) and transcription activator-like effectors nucleases (TALENs), this system has also been broadly used to perform “loss-of-function” and “gain-of-function” genetic studies to determine the roles of specific genes in cells or animals. The endogenous gene transcription could be modulated by means of manipulation of Transcription activator- like effectors (TALEs) or Zinc-fingers (ZFs) fused to transcriptional regulators (Sera, 2009; Chapdelaine et al., 2016). However, despite their efficacy, the exploitation of TALE and ZFs tools requires complex and time-consuming assembly protocols. Among the emerging applications of CRISPR-Cas9 based gene editing are techniques that incorporate a catalytically deactivated Cas9 (dCas9) fused to a protein domain to regulate transcription (Boettcher and McManus, 2015). Single guiding RNAs (sgRNA) target dCas9 fusion proteins to specific DNA segments such as promoters and enhancers, which may lead to activation (CRISPRa) or interference (CRISPRi) of transcription depending on the property of fused DNA cassette. Rosa26 knock-in constructs coupled with Cre/loxP recombination system have achieved conditional overexpression of exogenous genes in specific tissues, and also allow for site-specific integration of super large fragments (20–30 kb) (Hohenstein et al., 2008). However, this system generally needs complex knock-in operations and multiple-crossing schemes to generate desired mouse strains, which results into severe impact on the efficiency of the system. Bacterial artificial chromosome (BAC) based random transgenosis, as an alternative approach to overexpress a specific gene in animal model, can quickly and efficiently obtain the transgenic founder of mice. However, due to the integration of the gene cassette into a random locus, this technique may cause unwanted mutations and genes flanking the insertion site could be silenced unpredictably (Shinohara et al., 2007). Fortunately, the CRISPRa/dCas9 system possesses the potential to overcome these barriers described above. Numerous studies have demonstrated that the CRISPRa system is highly effective and easily tailorable to increase gene expression, or correct disease-causing mutations, with potential intriguing implications for therapy of human diseases (Matharu et al., 2019; Pignani et al., 2019; Savell et al., 2019). Nevertheless, plenty of room remains for further improvement and technical extensions on this system. The off-target mutagenesis was the initial concern about the therapeutic applications of CRISPRa system (Akcakaya et al., 2018). Another, but not the last, key question is how to efficiently deliver CRISPR-Cas9 into those tissues or cell types that are hard to infect or transfect (Ma et al., 2014). Fortunately, recombinant adeno-associated virus (rAAV) has so for become a most commonly used vehicle for CRISPR-Cas9 delivery because of its high efficacy, long-lasting transgene expression, less potential of immunogenic effect, and non-integration feature without random genomic insertion (Liu et al., 2017).

Harnessed as an effective genome editor, CRISPR-Cas9 has been broadly used in biomedical engineering of somatic cells (Liang et al., 2015; Cohen, 2018), and also been demonstrated as a promising tool to treat genetic disorders in model systems ranging from cells *in vitro* to animals *in vivo*. For example, it can restore dystrophin protein expression in cardiac and skeletal muscle by correcting mutations responsible for Duchenne muscular dystrophy (DMD) (Long et al., 2016; Nelson et al., 2016; Tabebordbar et al., 2016; Amoasii et al., 2018). In the case of cardiovascular disease, CRISPR gene editing can greatly reduce serum levels of PCSK9 and total cholesterol by targeting the PCSK9 gene in hepatocytes *in vivo* (Ding et al., 2014; Wang et al., 2016; Rossidis et al., 2018; Wang et al., 2018). Additionally, *in vivo* CRISPR-Cas9 gene editing prevents inherited retinal degeneration through selective ablation of S334ter mutation in a rat model of autosomal dominant retinitis pigmentosa (Bakondi et al., 2016). Moreover, CRISPR-Cas9 editing system is being explored for treating acquired diseases, such as cancer, HIV and hepatitis B (Seeger and Sohn, 2014; Soriano, 2017; Xiong et al., 2019). Nevertheless, successful correction of obesity-associated mutations *in vivo* via the CRISPR-Cas9 system has not yet been reported.

Single minded family basic helix-loop-helix transcriptional factor 1 (SIM1), a factor essential for neuronal differentiation and function of paraventricular nuclei (PVN) in the hypothalamus, plays a central role in food intake and energy homeostasis. Haploinsufficiency of Sim1, where one of the two gene copies is functionally lost, develops early-onset obesity with increased linear growth, hyperphagia, hyperinsulinemia and hyperleptinemia but no change in energy expenditure (Michaud et al., 2001). Hypothalamic Sim1 overexpression rescued diet-induced obesity due to reduced food intake (Kublaoui et al., 2006). Melanocortin-4 receptor (MC4R), a G protein-coupled receptor (GPCR) expressed in the hypothalamus, plays an important role in the appetite control and energy homeostasis (Huszar et al., 1997; Balthasar et al., 2005). MC4R expressed on glutamatergic Sim1 neurons in the PVN is both necessary and sufficient for the regulation of body weight (Balthasar et al., 2005; Xu et al., 2013; Shah et al., 2014). Haploinsufficiency of MC4R produces massive obesity in humans, while haplo-inactivation of MC4R in mice results in hyperphagic obesity (Huszar et al., 1997; Vaisse et al., 2000; Farooqi et al., 2003). In a very recent report, Matharu et al. utilized CRISPRa/dCas9 system to restore the expression of the haploinsufficient genes, Sim1 and MC4R, to physiological level in mouse models (Matharu et al., 2019). Different from canonical genome editing to correct disease causing DNA mutations, in this study, Matharu fused VP64, a universal moderate transcriptional activator to nuclease-deficient Cas9 (*dCas9*) to targets the promoter or enhancer, the non-coding genomic region of the remaining functional Sim1 gene to up-regulate endogenous gene expression. To examine the specificity and effectiveness of the approach, Matharu employed the transgenic animals carrying *spdCas9-VP64*, as well as rAAV mediated delivery of *pCMV-spdCas9-VP64* directly into the PVN of hypothalamus. In both cases, hypothalamic Sim1 expression restored to normal levels and thus rescued the obesity syndrome. Moreover, both hypothalamic transcriptome and ChIP-seq analysis showed that none of the Sim1 neighboring genes within a 500-kb window was differentially expressed, demonstrating the high specificity of this approach. Injecting rAAV-based CRISPRa into the hypothalamus of MC4R haploinsufficient mice similarly rescues the weight gain phenotype, further demonstrating the strength of this approach. This report presented us the first *in vivo* utilization of CRISPR-Cas9 system to rescues obesity due to haploinsufficient gene deficiency occurred in the central nerve system.

Moreover, this study also provides us with new strategy to treat obesity caused by abnormal gene expression: CRISPRa for haploinsufficient genes as reported in this study, or CRISPRi to treat diseases caused by pathogenic overexpression of a gene. Besides SIM1 and MC4R, the haploinsufficiency of proprotein convertase 1 (PCSK1), melanocortin 2 receptor accessory protein 2 (MRAP2) in the brain, iroquois homeobox 3 (IRX3) in fat or uncoupling protein 3 (UCP3) in mitochondria are also responsible for human obesity (Argyropoulos et al., 1998; Creemers et al., 2012; Asai et al., 2013; Zou et al., 2017). While overexpression of FTO, 11β-hydroxysteroid dehydrogenase-1 (11β-HSD-1), paternally expressed Mest (Peg1) in adipose tissue can also result in obesity (Kannisto et al., 2004; Rankinen et al., 2006; Church et al., 2010). These studies demonstrated that disrupting the expression of these genes may also rescue obesity by using CRISPRi technique. These results presented by Matharu et al. suggested that rAAV-mediated delivery of CRISPRa or CRISPRi targeting to cis-regulatory elements of these obesity-causing genes may be a better way, because this approach only regulates gene expression without modifying the coding region, thus expanding the options for choosing target and avoiding potential off-target mutations. While identifying and carefully characterizing the promoters and enhancers of these obesity-caused genes should be the primary issue that we need to address. Nevertheless, strict ethical argument and monitoring regulation are essential when carrying out this therapeutic strategy in human patients in the near future.
